# How Far Are We Justified in Anticipating Proximate Decay?

**Published:** 1875-09

**Authors:** E. D. Swain

**Affiliations:** Chicago.


### ARTICLE II.

### *How far are we Justified in Anticipating Proximate Decay?*

BY E. D. SWAIN, CHICAGO.

Read before the Illinois State Dental Society.

"Anticipate, to be before in doing, to do or take before another, take up beforehand or before the proper time,—to anticipate, supposes some ground or reason in the mind for considering an event as likely to happen, and in the practice of dentistry the question arises as to how far we are warranted in anticipating decay of the teeth, or in other words, how far are we justified in operating upon these organs for the prevention of anticipated decay?

It is a question of no small import to us as well as our patients, how we shall best forestall or prevent this destruction. That nearly all teeth are predisposed to decay, we are but too well aware. The conditions are so numerous, and the causes so obscure, that the difficulty of adopting any general rule must be obvious. Still I will endeavor to give my views, imperfect as they may be, as to when we are justified in anticipating decay, and the means to be employed to prevent it.

During the past fifteen or twenty years, comparatively little attention has been given to this subject by the profession at large. We have been satisfied to allow the destruction to proceed to a certain stage, and then use such means as in our power for arresting it; of late, however, the means of prevention has, to a certain extent, enlisted the attention of our profession.

In a paper of this character, it would seem proper to first consider, with our present knowledge, the character of teeth most likely to decay; which teeth and upon what part of them we are warranted in anticipating decay, as well as what means are to be used to prevent, in the largest number of cases, the commencement of decay of these teeth. Much to our discomfort, we are well aware that the deciduous teeth are, in our day, liable to decay; and the question frequently arises, whether in view of the fact that they must eventually be lost, any effort should be made to preserve them. I labor under the conviction that several important reasons exist why they should be, so far as possible, saved. The first is to prevent the suffering to which children are frequently subject, as the result of decayed teeth. The second, to avoid the injury to the general health which is the inevitable result of disease and loss of these teeth, and thirdly to prevent such irregularities as sometimes result from their premature loss. In my own practice, I do not in its fullest meaning, anticipate the decay of these teeth, but when upon frequent examination I detect evidences of decay upon proximal surfaces, I separate as freely and thoroughly as circumstances will admit of doing; and my experience, I think, warrants me in asserting that more of these teeth can be saved by this means than by any attempt at filling.

The anticipation of decay upon the mesial surfaces of the first permanent molars will not seem ambiguous to any person present, in case the second temporary molar is in its place; and the practice for prevention as given by Arthur in his work on "Treatment and Prevention," cannot fail to impress all as correct, and certainly no objection, from this point can be offered, viz: the cutting away of the distal surface of the second temporary molar in such a manner as to make a permanent space, and prevent any possible contact of the two teeth above the margins of the gums. The practice of some of allowing these teeth to go to destruction, with the avowed intention of extracting them at a later period believing thereby in all cases to secure more room, and an isolated condition of the remaining teeth cannot, in my estimation, be too emphatically condemned. If this end is accomplished, in the majority of cases it is only by the early removal of the tooth, before the other permanent teeth make their appearance. It must be admitted, however, that in isolated cases, good results are sometimes accomplished by the removal of the first permanent molar, viz: in cases of crowded arches, accompanied by an imperfect articulation of bicuspids. If however, the bicuspid teeth articulate perfectly, that is two upon one, in such a manner as to break joints, as it were, the cuspids and incisors are effectually prevented from spreading. I am convinced that better care of the first molars will enable us to save them, and in case it is desirable to sacrifice a tooth for the purpose of gaining more room for the other teeth, that it is a better practice to extract a bicuspid, from the fact that it is more difficult, in the majority of cases, to save these teeth by filling or filing. In fact, the more I read and practice Arthur's treatment for prevention of decay upon proximal surfaces, the stronger are my convictions that we have no more sure remedy. His advice as regards the prevention of decay upon these surfaces of all the permanent teeth as they are being erupted by cutting away the proximal surfaces of the adjoining temporary teeth, certainly cannot be condemned ; and we all know that during the process of shedding the temporary and erupting of the permanent teeth, the mouth is not in its best condition ; generally there is more or less a diseased condition of the soft tissues. The fluids are not unfrequently vitiated, and in a condition to expedite decay, and if we can by Arthur's or any other operation, preserve free from decay the permanent teeth through this period, we have greatly increased the chances of their being saved through life.

If decay attacks the central incisors on their mesial surfaces during the first or second years after eruption, I think we are fully justified in anticipating decay on the proximal surfaces of the other anterior upper teeth, and if permanent artificial spaces are not made between them, we may wedge them apart sufficiently to polish the proximal surfaces at points of contact. And I am of the opinion that by frequent wedging for this purpose another advantage may be gained: the arch is expanded, and the teeth are either permanently separated, or at least so much so that there will be no pressure at the point of contact. I am led to this belief from the fact that several adult people who once had crowded teeth, have informed me that the constant use of the floss silk or strips of india rubber, which I had advised for the purpose of cleansing the proximal surfaces, soon spread the teeth so much that no difficulty was experienced in the operation of cleaning them in this manner or with the pick.

Of the kinds of adult teeth most liable to decay, all are as well informed as myself. The white, chalky tooth, deficient in organic tissue, has perhaps given us all more thought and anxiety than any other class. With my present experience I am not prepared to give a general rule applicable for the treatment of such teeth. I have in mind one typical case of this class of teeth, which it may be well to mention here. Several of these teeth had been filled and re-filled by one of our most careful and efficient operators, upon their proximal surfaces, in such a manner as to preserve their contour; they however, persisted in decaying at their cervical margins. The lady applied to me about three years since, to have the bicuspids re-filled; in doing so considerable spaces were necessarily made, and in one or two instances superficial decay was removed; being one who cares for her teeth, I was called upon to examine them frequently, and in the meantime the separation of teeth as a means of prevention had been brought to our notice, and the results were so flattering where I had separated for filling, as well as where superficial decay had been removed, that I have since made permanent spaces between nearly all her teeth; and although three years have elapsed since the first operations, I must acknowledge my pleasure at the result as manifesied. I will not occupy your time by reporting other cases treated in like manner, though I have adopted it in all cases of similar character, with uniform success so far as I am able to judge.

There is another class of teeth which has given me much anxiety, viz: the short, round (or square) yellowish tooth, standing close together and very regular; the kind of teeth so liable to mechanical abrasion upon their grinding surfaces, not very liable to be attacked by caries, except upon their proximal surfaces. In my own practice, I do not hesitate to use the disks, making free and permanent spaces, as advised by Arthur, and I have yet to see the teeth thus separated which have caused me to regret the means used.

We are also warranted in anticipating proximal decay where the teeth are of the long, flat order, (such as are *almost* invariably to be met within persons of a consumptive diathesis,) standing, as they do, in close contact, and especially in cases where the fluids of the mouth are in that condition which experience has taught us is conducive to decay, especially where they do not receive the most scrupulous care. Further, in those cases of irregularity, where the labio-distal surface of one tooth is covered by the palato-mesial surface of its neighbor, or *vice versa.* In these cases, I know of no method so effectual as to make such a separation as to leave the teeth permanently apart, unless it be for the second class mentioned, the operation of regulating. Every one present, of extended experience, cannot fail to call to mind such cases treated as above, in which the decay has been permanently prevented or arrested.

That class of teeth presenting the bell-shaped crowns, forming by their peculiar structure a triangular space, bounded by the teeth above the point of contact on two sides, with the gum as the base of the triangle, are especially liable to decay upon their proximal surfaces; and while this class of teeth are easily kept clean, if the possessor gives them the necessary attention, still we often see these pockets filled with the food of several days, preceding an examination;—with this class of teeth, more objections may be raised to the Arthur treatment than with those above mentioned, still I am under the impression that it may be profitably used here, by chiseling away the bulge, leaving a

V shaped space opening into the mouth, thereby reducing the point of contact to its minimum. While the greatest enemies of the Arthur method admit that perfect cleanliness would save many teeth from decay upon their proximal surfaces, still they contend that self-cleansing surfaces cannot but prove destructive, simply because the enamel has been removed. They ignore entirely the vital change so well established, which takes place in exposed dentine for its own protection as well as that of the pulp within. They virtually dispute such authorities as Tomes, Salter, and others whose investigations have proved beyond question, that not only in cases of decay and abrasion, but where the enamel has been artificially, so to speak, removed, that the dental tubuli are filled with lime salts, the result of which is a dense surface, resisting the ravages of decay, and in cases of abrasion, chemical or mechanical, capable of receiving a polish equal to the enamel itself, and almost, if not quite capable of resisting the attacks of decay or caries as well as the enamel itself. Mr. Salter also informs us (and who that has examined into this matter will not agree with him) that dentine of repair, as he terms it, is invariably deposited upon the inner ends of the injured tubuli, whether the injury arises from decay, abrasion or fracture; if this be true, why are we not justified in expecting these changes when the file has been used for the removal or prevention of decay? For my part, I believe my own observation warrants the acceptance of this theory as correct.

Investigation is every day proving to us, that the teeth are not so void of vitality as we were once led to believe, and that under proper conditions they do possess the vital power to resist the attacks of disease, as well as, in a measure, to repair themselves when injured. Notwithstanding we are informed on page 442 Missouri Dental Journal, Dec., 1874, that this vital force is a positive disadvantage, and that teeth possessing the greatest amount of it are the ones most liable to be attacked by disease; in other words, we are told that what we have always conceded to be a pathological condition, is on the contrary the highest form of life in these organs. Webster gives us the definition of vital—"as belonging to life, either animal or vegetable, as vital energies, powers, &c.; as contributing to life, necessary to life, as vital air, or blood; containing life, living, or being the seat of life. Being that on which life depends, capable of living or in a state to live." I understand that disease of a tissue, either animal or vegetable, portends death, unless the disease be removed,—and who has not learned that caries and exalted sensitiveness are diseases, and portend death to the teeth thus affected. I do not understand that great vital force, as we use the term, is ever the cause of disease. If however, it so proves, we will accept the corn argument upon page 451, and recommend at once that upon our return home, each dentist present proceed to devitalize all the teeth in the mouths of patients presenting themselves for treatment.

In recommending Arthur's method for prevention of decay of the teeth, I do not wish to be understood as advising the indiscriminate use of the file or disk; good judgment is necessary, more, perhaps, than in deciding when and how to fill a tooth. When I first read this book, I laid it down with perhaps as much prejudice and dissatisfaction as any one present. I have since read it in a more careful manner, have observed carefully all teeth which came under my eye from which the enamel had been removed, from any cause, and the result is, I am becoming converted to a careful, discriminate use of his method. If wrong, I am anxious to be set right, and hope to learn much from those present, upon this subject.

